# Probing Structural Features and Binding Mode of 3-Arylpyrimidin-2,4-diones within Housefly γ-Aminobutyric Acid (GABA) Receptor

**DOI:** 10.3390/ijms12096293

**Published:** 2011-09-23

**Authors:** Qinfan Li, Lihui Zhang, Zhi Ma, Xiangya Kong, Fangfang Wang, Hong Zhang, Yonghua Wang

**Affiliations:** 1 College of Veterinary Medicine, Northwest A & F University, Yangling 712100, Shaanxi, China; E-Mails: liqinfan@yahoo.com.cn (Q.L.); zhanglihui200701@163.com (L.Z.); 407147075@qq.com (X.K.); 2 College of Life Science, Northwest A & F University, Yangling 712100, Shaanxi, China; E-Mails: mshappy1986@126.com (Z.M.); yu100288@163.com (F.W.); zhtear99@163.com (H.Z.); 3 Center of Bioinformatics, Northwest A & F University, Yangling 712100, Shaanxi, China

**Keywords:** 3-arylpyrimidin-2, 4-diones, GABA receptor, 3D-QSAR, homology modeling, molecular dynamics simulation, molecular docking

## Abstract

In order to obtain structural features of 3-arylpyrimidin-2,4-diones emerged as promising inhibitors of insect γ-aminobutyric acid (GABA) receptor, a set of ligand-/receptor-based 3D-QSAR models for 60 derivatives are generated using Comparative Molecular Field Analysis (CoMFA) and Comparative Molecular Similarity Index Analysis (CoMSIA). The statistically optimal CoMSIA model is produced with highest *q^2^* of 0.62, *r^2^*_ncv_ of 0.97, and *r^2^*_pred_ of 0.95. A minor/bulky electronegative hydrophilic polar substituent at the 1-/6-postion of the uracil ring, and bulky substituents at the 3′-, 4′- and 5′-positions of the benzene ring are beneficial for the enhanced potency of the inhibitors as revealed by the obtained 3D-contour maps. Furthermore, homology modeling, molecular dynamics (MD) simulation and molecular docking are also carried out to gain a better understanding of the probable binding modes of these inhibitors, and the results show that residues Ala-183(C), Thr-187(B), Thr-187(D) and Thr-187(E) in the second transmembrane domains of GABA receptor are responsible for the H-bonding interactions with the inhibitor. The good correlation between docking observations and 3D-QSAR analyses further proves the model reasonability in probing the structural features and the binding mode of 3-arylpyrimidin-2,4-dione derivatives within the housefly GABA receptor.

## 1. Introduction

As ligand-gated chloride channels that belong to the Cys-loop receptor family, ionotropic γ-aminobutyric acid (GABA) receptors are widely distributed throughout the vertebrate and invertebrate central nervous system where they predominantly mediate the effects of inhibitory neurotransmitter GABA [1–3]. The ionotropic GABA receptor exists as pentameric membrane protein [4], with each subunit consisting of an *N*-terminal extracellular domain, four α-helical transmembrane (TM) domains, where the TM2 domain lines the integral chloride channel, and a large intracellular loop between TM3 and TM4 [5]. In insect, researchers have cloned three kinds of subunits of ionotropic GABA receptors: Rdl (resistant-to-dieldrin), LCCH3 (ligand-gated chloride channel 3) and GDR (the GABA_A_ and glycine receptor-like subtype of *Drosophila*), wherein the Rdl is the unique subunit known to form functional GABA-gated channel, suggesting its tendency to be a major subunit of the insect GABA receptor [5,6].

Insect GABA receptors are the molecular targets of non-competitive antagonist insecticides, such as fipronil and picrotoxinin, which have been widely applied in pest insect control by exerting their effects on insect GABA receptors by way of decreasing the Cl^−^ influx into the neurons [7–11]. The polychlorocycloalkane lindane and dieldrin are classical chlorinated insecticides, which have been banned due to their persistence in the environment and their resistance to the insects [6]. Fortunately, phenylpyrazole fipronil, a major replacement insecticide acting at the GABA receptor, has been successfully developed showing excellent selectivity and activity for insect chloride channels, as well as low mammalian toxicity and low persistence in environment [12]. However, due to its extensive or inappropriate use nowadays, fipronil has exhibited undesirable threats on birds [13,14] and aquatic organisms [15]. Therefore, the development of new GABA chloride channel insecticides as an alternative is still urgent for the pest insect control recently.

On GABA receptor, the binding assays of site-directed mutants indicate that amino acids Ala2′, Thr6′ and Leu9′ at the TM2 region of GABA receptor might be involved in the interactions with those non-competitive antagonists (NCAs) [10,12,16]. However, some other evidences also suggested that the structurally diverse NCAs might have distinct binding modes in GABA receptor [2,17,18]. For instance, fipronil might bind to the TM3 while picrotoxinin and dieldrin bind to the TM2 region of GABA receptor [2]. However, it still remains ambiguous whether all NCAs with diverse structures interact at the same or an overlapping site, or multiple sites within the channel pore [16,19]. Recently, the replacement of the channel-lining A2′ amino acid on the cytoplasmic side (Ala2′ to Ser in the *D. melanogaster* Rdl) was also found to confer resistance to diverse insecticides, such as fipronil, dieldrin, picrotoxinin and picrodendrin-O [1,6,20]. A radiolabeled-ligand-binding assay showed that Thr6′ mutation could completely abolish the binding ability of ethynylbicycloorthobenzoate (EBOB) [16]. These mutations potentially perturb the gating kinetics and decrease the binding potency of NCAs.

Besides the experiments probing the structural features of NCAs interacting with GABA receptor, *in silico* methods, such as the three-dimensional quantitative structure-activity relationship (3D-QSAR) analysis, have been introduced to analyze several kinds of NCAs, such as endosulfan [19], bicyclophosphates [19], and 1-phenyl-1H-1,2,3-triazoles [21]. The specific structural and electrostatic features defined by the comparative molecular field analysis (CoMFA) and comparative molecular similarity indices analysis (CoMSIA) are found to be essential for enhancing the binding of these NCAs in the GABA receptors [21]. In addition, hydrophobicity, a possible factor controlling the transport behavior of compounds, is also significant in governing variations in insecticidal activity [19].

More recently, to quest new GABA chloride channel insecticides, a series of 3-arylpyrimidin-2,4-diones (APDs) have been developed exhibiting equivalent efficacies to fipronil by *in vitro* GABA assay [9]. The *in vivo* experiments also showed that APDs not only excellent control against the southern corn rootworm in the greenhouse but also are insecticidal against the plant hopper, rice leafhopper, twenty-eight-spotted lady beetle and two-spotted spider mite with no method of analysis disclosed [9]. As mainly concerns are taken into account with the potency of APDs, several questions about APDs still remain to be clarified: (1) what are the structural features of APDs indispensable for improvement of the potency? (2) how do APDs interact with the insect’s GABA receptor at a molecular level? (3) what is the similarity/difference of the binding sites between these compounds and other reported NCAs?

Therefore, to answer the above questions and to explore these key structural features impacting the potency of APDs, 3D-QSAR analyses using the CoMFA and CoMSIA methodologies are applied in this work on a group of APDs analogues as GABA receptor ligands. In addition, homology modeling, molecular docking and molecular dynamics simulation are also performed to elucidate the probable binding modes of these inhibitors within the GABA receptors. The good consistency between 3D contour maps and the topographical features of the binding sites of APDs leads to our identification of the developed models, which might provide useful information for further guiding the structural modification and design of new potential APDs insecticides.

## 2. Results and Discussion

### 2.1. Statistical Analysis

**Figure 1:**
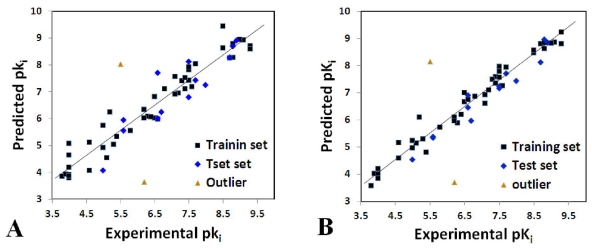
Plot of the predicted p*K*_i_ *versus* the experimental p*K*_i_ values for the models based on the training (filled black squares) and test (filled blue rhombuses) sets. (**A**) CoMFA, (**B**) CoMSIA. The solid line is the regression line for the fitted and predicted bioactivities of training compounds. The outliers in test set are shown in orange triangles.

Ligand- and receptor-based alignment methods were applied to produce the models for CoMFA and CoMSIA analysis. In terms of statistical parameters, the *q^2^* (0.60 and 0.62), *r^2^*_ncv_ (0.94 and 0.97) and *r^2^*_pred_ (0.82 and 0.95) values obtained from ligand-based models (Table 1) suggest that these models had good statistical correlation and predictive capacity. Additionally, the good consistency of the ligand-based models between the 3D-QSAR maps and docking results also indicates the robustness and reasonability of the models. However, compared with the ligand-based models, the receptor-based ones generate the low *q^2^* (0.34 and 0.55) and *r^2^*_ncv_ (0.81 and 0.85) values (Table 1), which indicate the bad predictive capacity of the receptor-based models. Moreover, the receptor-based alignment was more scattered than the ligand-based one (as shown in Figure 1). Thus, the models derived from ligand-based alignment are better than those derived from receptor-based one.

The CoMFA model describing APDs inhibition using both S and E fields produces a *q^2^* = 0.60 and an *r^2^*_ncv_ = 0.94 using six optimum components with an F-statistic value (*F* = 90.71) and a standard error of estimate (SEE = 0.48), which signify a good statistical correlation and predictive capacity of the model (*q^2^* > 0.5) [22]. The corresponding contributions of S and E fields are respectively 57.3%, and 42.7%, indicating that the S field has a greater influence than the E field in inhibition potency.

The external test set of 15 molecules was employed with the purpose of testing the stability and predictive ability of the constructed CoMFA model. Compounds 14 and compound 21 regarded as outliers were omitted from the final analysis, since their differences between the experimental and predicted p*K*_i_ values were more than one logarithmic unit. A bulky higher hydrophobic substituent of compound 14 and hydrogen substituent of compound 21 at 1-position of the uracil ring might account for their outlier status. The predicted correlation coefficients (*r^2^*_pred_) for the CoMFA model is 0.82, proving its proper capability for prediction of the test inhibitors. The correlation between the predicted and experimental p*K*_i_ values of all compounds in the CoMFA model is shown in Figure 1A.

In comparison to CoMFA, CoMSIA methodology using the same standard has an advantage of exploring more field descriptors for the APDs inhibition. In the CoMSIA model, statistical parameters show that S, E, H, D and A features significantly influence the activity of the compounds. The *q^2^* (0.62), *r^2^*_ncv_ (0.97), *SEE* (0.32) and *F* (126.18) values obtained from the model indicate a good predictive capacity and internal consistency. In addition, the percentages of the variance explained by S, E, H, D and A descriptors are respectively 0.139, 0.338, 0.383, 0.059 and 0.081, implying that the hydrophobic field which is not included in the CoMFA model is important for explaining the potency of the molecules. Furthermore, the CoMSIA model possesses better prediction with high *r^2^*_pred_ (0.95) than CoMFA model under the same outliers. The correlation between the predicted activities and experimental p*K*_i_ values is displayed in Figure 1B. Overall, CoMSIA model is more reliably applied than the CoMFA model in description of the APDs inhibition and the design of new inhibitors.

### 2.2. 3D-QSAR Contour Maps

To visualize the information content of the derived 3D-QSAR models, CoMFA and CoMSIA contour maps were generated by plotting the field energies at each lattice point as the percentage of the contribution to the CoMFA or CoMSIA equation, which were calculated as the scalar results of the coefficient and the standard deviation. The colored polyhedra in the map surrounds all those lattice points where the 3D-QSAR models strongly associate the changes in the compounds’ field values with those changes in the biological potency. To aid in visualization, the highest active compound 58 (p*K*_i_ = 9.3) is overlaid in the contours as a reference.

**Figure 2:**
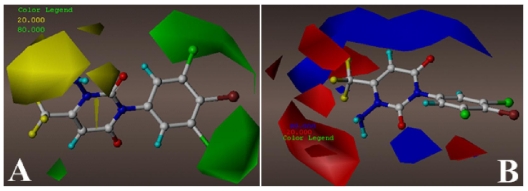
Stdev*coeff (**A**) steric, (**B**) electrostatic contour maps of ligand-based optimal CoMFA model. The color code is as follows: (**A**) green and yellow contours indicate favorable and unfavorable bulky groups, respectively; (**B**) blue and red contours indicate favorable and unfavorable electropositive groups, respectively. The compound 58 in ball and stick is displayed as a reference. (Yellow atom: fluorine; red: oxygen; blue: nitrogen; green: chlorine; brown: bromine).

The CoMFA contour maps of steric and electrostatic fields are shown in Figures 2A and 2B, respectively. In Figure 2A, two large regions of green contour close to the 3′-, 4′-, 5′-positions of the benzene ring indicate that bulky substituents are preferred in those positions. Compounds 7 (3′,4′,5′-triCl) and 22 (3′,4′,5′-triCl) completely integrate with the green contours, apparently responsible for their higher potencies than the corresponding analogs (compounds 1–4, 12, 40, 42, 43, 47, 49) without -Cl at all these positions. Additionally, a small green contour near the -CF_3_ substituent at 6-position of the A ring explains why the binding affinity of compound 35 (6-propyl) is higher than that of the corresponding compound 29 (6-CH_3_). Some regions of yellow contour are noted near the amino substitution at 1-position of ring A, suggesting that a bulky group appeared there would decrease the activity. This is in agreement with the fact that the substituent of compound 20 is too bulky to fit into yellow region and thus is responsible for its weak inhibitory activity.

Due to the strong electron-withdrawing ketone substituent, two large regions of blue contour above and below ring A, corroborate the favorability of partial positive charge in these regions. A small blue contour near 6-position of ring A suggests that an appropriate electropositive substituent at the position may lead to an increase in the binding affinity. This may explain why compound 29 shows decreased potency compared with compounds 24 (6-isopropyl) and 30 (6-butyl) by replacing the isopropyl or butyl group with an -CH_3_ group. Besides, a large red polyhedral near the amino substituent of ring A suggests that electronegative substituents are well tolerated there. The fact that the -CH_2_CF_3_ and -CH_2_CN substituents at 1-position of ring A of compounds 15 and 17 respectively are more beneficial to the activities than the -CH_2_COCH_3_ group of compound 16 is just the case. Other red contours surrounding the -CF_3_ substituent of ring A allow us to speculate that electronegative substituent would increase the binding affinities. The good inhibitory potencies of molecules 25(-CF_2_CF_2_CF_3_) and 33(-CN) are due to the orientations of these substituents towards the above red contours. In addition, a small red area also appears above the meta-position of the benzene ring. The above information offered from the CoMFA contour maps is helpful for us to further explore the inhibitor-GABA receptor interactions.

**Figure 3:**
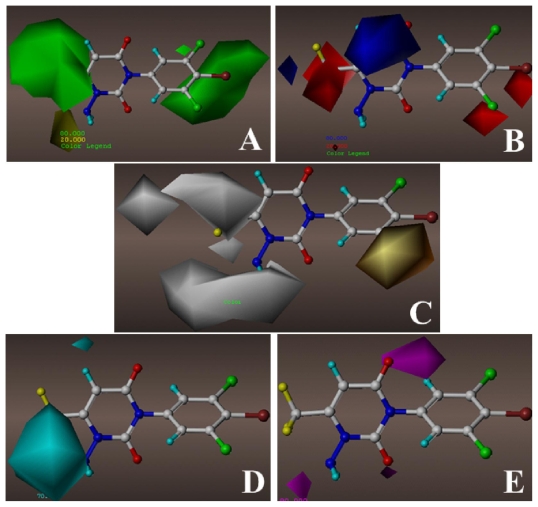
stdev*coeff (**A**) steric, (**B**) electrostatic, (**C**) Hydrophobic, (**D**) H-bond donor and (**E**) H-bond acceptor contour maps of ligand-based optimal CoMSIA model. The color code is as follows: (**A**) green and yellow contours indicate favorable and unfavorable bulky groups, respectively; (**B**) blue and red contours indicate favorable and unfavorable electropositive groups, respectively; (**C**) orange and white contours indicate favorable and unfavorable hydrophobic groups, respectively; (**D**) cyan contours indicate favorable H-bond donor groups; (**E**) magenta contours indicate favorable H-bond acceptor groups. The compound 58 in ball and stick is displayed as a reference. (Yellow atom: fluorine; red: oxygen; blue: nitrogen; green: chlorine; brown: bromine)

With respect to the steric and electrostatic contour maps, the information of contour maps obtained from the optimal CoMSIA model (Figures 3A and B) is mostly similar to that derived from above CoMFA one except for the different distribution ranges. The hydrophobic interaction in the CoMSIA model is represented in Figure 3C. Two large orange contours surrounding the meta-position of benzene ring demonstrate the importance of the -Cl group for the binding activity. Meanwhile, a super large white contour map sets around the 1-position of ring A, suggesting the favor of hydrophilic groups for potency. When comparing compound 23 (-NHCOCH_3_) with 16 (-CH_2_COCH_3_), as well as 22 (-NH_2_) with 7 (-CH_3_), it can be easily found that their activity discrepancies also attribute to this white contour. The -CF_3_ group of ring A fills into several large white contours, indicating that hydrophilic groups are essential for the inhibitory activity. Replacing -CH_3_ group (29) by -CN group (33) at C-6 leads to increased activity.

The CoMSIA contour maps of the hydrogen-bond donor and acceptor fields are shown in Figures 3D and 3E, respectively. In Figure 3D, a large cyan contour appears enclosing the amino substituent of ring A, which suggests that the amino group acts as an H-bond donor by forming H-bond with the residue of the binding site. The cyan contour implies that an optimum H-bond donor group is expected to increase the activity, which agrees well with the fact that compounds 50–60 with the amino group at the N-1 show significantly increased potency compared to the analogous compounds 7 and 13–21. In the H-bond acceptor contour map, two magenta contours near two ketone groups of ring A indicate the importance of H-bond acceptor group for the binding activity. In addition, the presence of another magenta contour near 6-position of ring A reveals the extreme importance of the -CF_3_ group in 6-position.

**Figure 4:**
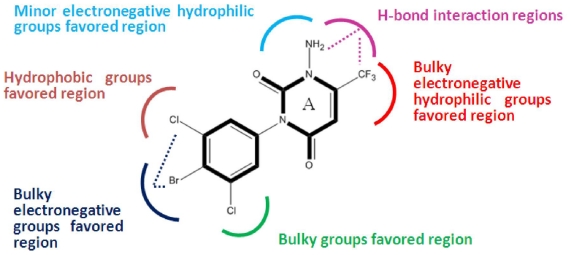
Structural features obtained from ligand-based optimal CoMSIA model. The common substructure of model compound 58 is shown in bold.

The structural features obtained from above CoMSIA models are depicted in Figure 4. Changes in the binding affinity of the inhibitors could be rationalized by modifying the inhibitors based on these key structural features: (i) Electronegative hydrophilic polar substituents with minor/bulky size like -NH_2_/-CF_3_ groups are preferential at 1-/6-positions of ring A respectively, which can enhance the activity of the inhibitor by providing a hydrogen bonding interaction with the protein target; (ii) Bulky electronegative substituents at 3′- and 4′-positions with a simultaneous big group at 5′-position of the benzene ring are beneficial for the enhanced potency of the inhibitors.

### 2.3. Homology Modeling and Molecular Dynamics Simulation

**Figure 5:**
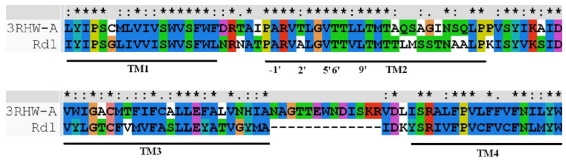
Alignments of the sequences of the TM domains between the Rdl subunit of housefly GABA receptor and the 3RHW chain A template. Different types of amino acids are depicted in different colors. The four transmembrane domains (TMDs) of Rdl subunit are indicated with bold lines and the positions of the 2′, 6′ and 9′ residues in TM2 of Rdl subunit are also indicated. “*”, identical residues; “:”, conserved substitutions; “.”, semi-conserved substitutions.

**Figure 6:**
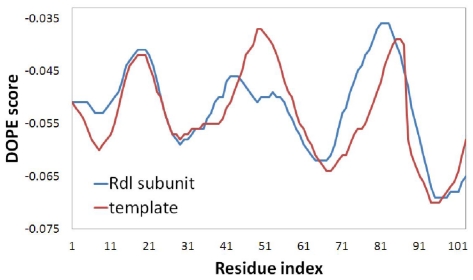
Discrete optimized potential energy (DOPE) score profiles of the template and modeled Rdl subunit.

**Figure 7:**
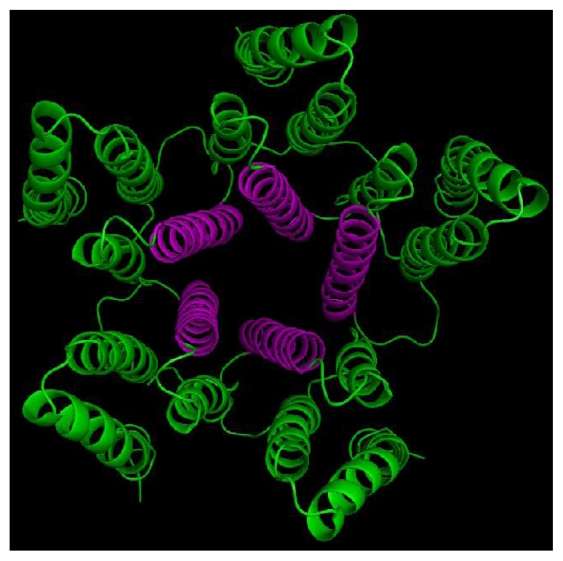
The final refined TM domains of the housefly GABA receptor shown in cartoon representation. Green: TM1, TM3, TM4; magenta: TM2.

Although the subunit composition of native insect GABA receptor is unknown, it has been confirmed that Rdl subunit can be expressed to form functional homo-oligomeric receptor [6], which has been used by Ian McGonigle to determine the critical features of GABA receptor agonists [23]. The modeling of Rdl subunit was carried out based on the template of glutamate-gated chloride channel receptor in this work. It is known that both the GABA receptor and glutamate receptor are members of the superfamily of Cys-loop ligand-gated ion channels, and their subunits both contain four TM regions. Since the Rdl subunit of housefly GABA receptor in the TM domains has a high level of sequence identity (47%) with the template, especially in the TM2 domain (62%). The multiple sequence alignment of TM domains between the Rdl subunit with the chains of glutamate receptor (shown in Figure 5) demonstrates that the TM domains between the GABA receptor and glutamate receptor are relatively conserved, suggesting that glutamate receptor structure is an appropriate template for the modeling of the TM domains of GABA receptor. During the modeling process, the relative stability of the model was evaluated by the discrete optimized potential energy (DOPE), which scores of the template and the optimal modeled Rdl subunit are shown in Figure 6. Compared with the DOPE scores of the template, every residue of the model shows similar energy, suggesting that the loop refinement is not necessary for the optimal model. The overview of the final modeled housefly GABA receptor is shown in Figure 7.

**Figure 8:**
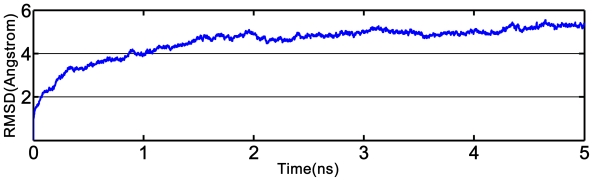
Plot of the root-mean-square deviation (RMSD) of refined GABA receptor structure *versus* the MD simulation time.

To obtain the “real” bioactive conformation, 5 ns molecular dynamic simulation was employed to make the structure of the refined receptor more reasonable by considering both the impacts of the receptor flexibility and the effects of water solvation on the complex. The dynamical picture of the conformational changes is shown via the root-mean-square deviation (RMSD) in Figure 8. The RMSD of the trajectory with respect to the initial structure ranges from 1 to 5.5 Å, and reaches a plateau about 5.3 Å after 3 ns, indicating that the structure conformation thereafter is stable and reliable for the subsequent docking study.

### 2.4. Docking Analysis and Comparisons with 3D-Contour Map

**Figure 9:**
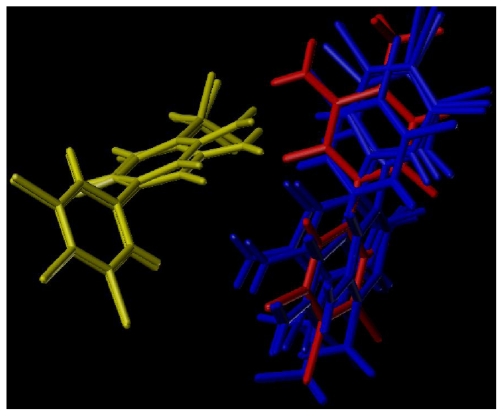
The structural conformations of the top 10 scored poses. Blue: orientation I; red: orientation II; yellow: orientation III.

**Figure 10:**
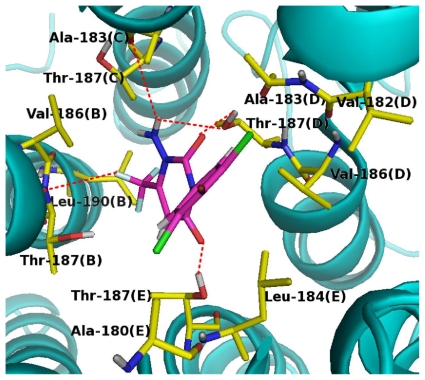
Docking of model compound into the GABA binding sites. Model compound and residues within 4.5 Å are shown as stick representation. Hydrogen bonding interactions are shown as red dashed lines. Yellow and magenta: carbon; red: oxygen; blue: nitrogen; cyan: fluorine; green: chlorine; brown: bromine.

Docking, which plays an important role in the rational design of drugs, is frequently used to predict the binding orientation of drug candidates to their protein targets (active sites) and also to predict the binding affinity of the molecules in turn [24]. In the present study, dockings of all compounds into the housefly GABA receptor were carried out to find the optimal orientations of the compounds. Based on previous studies [10,12,16,25], we chose the T6′ (Figure 5) residue of Rdl subunit as the active site in the chloride ion channel to conduct the docking of the most potent compound 58. The analyze of the top 10 scored (4.49–2.23) docking poses (as shown in Figure 9) shows that the top 5 scored poses display similar orientations (shown as orientation I) while the seventh and tenth scored poses show the opposite orientations (orientation II). From these poses, the structural conformation of the highest scored pose is adopted since it has the highest binding free energy and also is well consistent with the QSAR contour maps as mentioned below in docking analysis. More importantly, the binding sites of the docking model are in accordance with site-directed mutation assays [5,12,16], which imply that amino acids A2′, T6′ and L9′ (Figure 5) in TM2 region play key roles in the inhibitor recognition. However, the sixth and ninth scored conformations have a different orientation (orientation III) with other poses, which results in changing the steric environment of the compound to decrease the score and create the weak binding pattern. Combined, the obtained structural conformation with compound 58 is considered to be the optimal docking model (as shown in Figure 10).

In Figure 10, the key residues that form the binding pocket of GABA receptor are Val-186(B), Thr-187(B), Leu-190(B), Ala-183(C), Thr-187(C), Val-182(D), Ala-183(D), Val-186(D), Thr-187(D), Ala-180(E), Leu-184(E) and Thr-187(E), which are located in the TM2 regions of four segments. The model compound is anchored in the binding pocket via several H-bonds. The amide of ring A is capable of making similar H-bonds with the backbone carbonyl of Ala-183(C) (-NH•••O, 3.89 Å, 108.3°) and the side chain hydroxyl group of Thr-187(D) (-NH•••O, 3.60 Å, 75.8°) as H-bond donors, which are consistent with the cyan contours at the H-bond contour maps (Figure 3D) near this position. These H-bonding interactions of the amide indicate that the potential H-bond-donating NH plays a critical role in the binding activity. Additionally, ketone groups at 2- and 4-positions as H-bond acceptors prefer to construct H-bonds with the side chain hydroxyl groups of Thr-187(D) (-OH•••O, 3.54 Å, 97.4°) and Thr-187(E) (-OH•••O, 3.38 Å, 162.2°), respectively. It is remarkable to note that the -CF_3_ group of ring A forms a weak H-bond (5.60 Å) with the backbone NH of Thr-187(B). These observations as mentioned above are in full agreement with the H-bond accepter contour map (Figure 3E). As an outlier in the ligand-based model, compound 21 shows little different orientation in the docking pocket, and is also considered to be an outlier in the structure-based model. For this compound, the absence of H-bond-donating substituent at 1-position of the ring A only results in the H-bond formation between its ketone groups and the side chains of Thr-194(C) and Thr-194(D) in the binding pocket, indicating the weak binding pattern of this compound. This clearly demonstrates why compound 21 is so low in activity compared with compound 58 (p*K*_i_ = 9.3).

The docked model shows that the amide of ring A fits nicely into a relatively large pocket composed by Ala-183(C), Thr-187(C) and Thr-187(D), indicating that the steric interaction in this hydrophilic region is deleterious to the inhibitory activity. However, compared with compound 58, the large -CH_2_C(CH_3_)_3_ substituent of compound 14 leads to steric bump with Thr-194(A) and Thr-194(E) residues and disturb the optimal position of the compound in the pocket, which is one reason of the poor activity of compound 14 (p*K*_i_ = 6.2, an outlier in the model). This result is well consistent with the steric contour map of the CoMSIA model, which owns a yellow contour (Figure 3A) at the same location. Residues Val-186(B), Thr-187(B) and Leu-190(B), not only form a sufficient hydrophilic room to accommodate a large hydrophobic -CF3 substituent, but also are suitable for electrostatic interactions with negatively charged -CF3 group, which indicates that a bulky and electronegative substituent at this position would be favorable to the activity. Figure 10 also shows a comparatively large empty space formed by hydrophobic residues Val-182(D), Ala-183(D), Ala-180(E) and Leu-184(E) around the benzene ring, suggesting that a suitable bulky electronegative substituent on the benzene ring is needed and would have a favorable interaction with the backbone NH of Val-182(D) and Ala-183(D). The blue contour above the A ring (Figure 3B) implying an electropositive favorable region, is consistent with the appearance of Val-186(D) and Thr-187(D) surrounding this area. These observations are also demonstrated by the presence of several green and red contours (Figures 3A, 3B and 3C) around these regions. Overall, the results indicate that the binding model obtained from the docking analysis is reasonable and suggest that the amide and ketone groups play major roles in the high binding potencies of these inhibitors of the GABA receptor.

### 2.5. Binding Mode for APDs in the Housefly GABA Receptor

To identify the binding sites of NCAs in GABA-gated chloride channels, some mutagenesis studies show that the amino acids include Ala2 (A2′), Leu3 (L3′), Thr6 (T6′) and Leu9 (L9′) (index numbers for the TM2 region) in the channel lumen are responsible for the interactions of NCAs [10,12,16,25]. Correspondingly, as illustrated in our docking model of the complex (Figure 10), the model compound fits into the putative binding pocket lined by five TM2 segments in the channel. The analyses of H-bond interactions in the docking complex indicate that A2′ (Ala-183) and T6′ (Thr-187) (Figures 5 and 10) play key roles in the inhibiting interaction of APDs with housefly GABA receptor, which is consistent with earlier mutagenesis study in the β3 homopentamer that A2′ and T6′ mutations reduce or destroy the NCA radioligand binding [16]. In addition, A-1′ (Ala-180), 1′V (Val-182), A2′ (Ala-183), L3′ (Leu-184), V5′ (Val-186), T6′ (Thr-187) and L9′ (Leu-190) residues without H-bonding contacts with the compound, are likely to contribute to the APDs’ binding by maintaining the structural integrity of the binding site, or mediating the local conformational movements near the binding site.

However, the binding sites for APDs focusing on the A2′ and T6′ in TM2 region are shared as two identical key sites with other antagonists, such as fipronil, picrotoxinin and EBOB [5,12,16]. Although the amino acids V5′ and L9′ of Rdl subunit are indirectly involved in the binding of APDs, the amino acids at 5′ and 9′ positions in TM2 region are also two potential sites responsible for the binding of NCAs, while the fipronil-related NCAs [10,12] are in hydrophobic contact with I5′ and L9′ of β_3_ subunit. Whereas, compared with fipronil [2], S17′ residues within the channel does not exist in the interaction of APDs. In summary, structurally different classes of NCAs bind to two identical sites and two potential sites in different or overlapping orientations within the channel pore of the GABA receptor, except for a few NCAs with multiple sites.

## 3. Materials and Methods

### 3.1. Dataset

Presently, sixty 3-arylpyrimidin-2,4-diones derivatives acting on housefly GABA-gated chloride channel collected from reference [9] were used as dataset with a wide activity ranging from 3.8 to 9.3 (p*K*_i_ values), which was then divided into a training set and a test set in a ratio of 3:1 based on adequate coverage in terms of both the inhibitory activity and the structural diversity. The training set was used to construct 3D-QSAR models and the test set (asterisked molecules in Table 1) was used for the model validation. Table 2 shows the structures and biological data (p*K*_i_) of all compounds.

### 3.2. Molecular Modeling and Alignment

**Figure 11:**
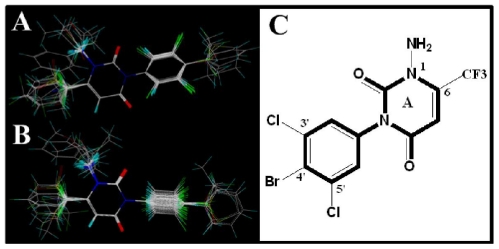
The alignment models of all compounds: (**A**) ligand-based alignment; (**B**) receptor-based alignment; (**C**) The common substructure of compound 58 for alignment is shown in bold.

Molecular modeling and alignment were carried out by using the SYBYL package (Tripos Associates, St. Louis, MO, USA). The energy optimization of each compound with Gasteiger-Huckel charges was performed using the Tripos force field [26] with a distance-dependent dielectric and Powell gradient algorithm with a convergence criterion of 0.05 kcal/mol. To obtain the optimal 3D-QSAR statistical model, two different alignment rules, *i.e*., the ligand- and receptor-based alignments were adopted to identify the most efficient alignment approach for this data set. In the first approach, all compounds were aligned to the most potent compound 58 by the Align-Database function using the common substructure (Figure 11). For the second method, the bioactive conformations of all compounds were firstly derived from docking and then processed using the first method. The resulting ligand-/receptor-based alignment models are shown in Figure 11.

### 3.3. Homology Modeling and Molecular Dynamics Simulation

Protein sequence of Rdl subunit in *Musca domestica* (house fly) GABA receptor (ID AAC23602) was retrieved from the NCBI web site [27]. Before homology modeling, the sequence was edited to remove the extracellular region and residues in the loop between transmembrane (TM) domains 3 and 4 due to unavailable original template and their no contribution to the binding of NCAs. The TM domains were modeled using MODELLER software [28] with the template of X-ray crystal structure of glutamate-gated chloride channel receptor (PDB code 3RHW, 3.26 Å) based on the sequence and structure comparison of the TM domains of GABA-gated chloride channel receptor. The multiple sequence alignment of TM domains between the Rdl subunit with the chains of glutamate receptor was carried out using the ClustalW program [29]. To evaluate the fold of the optimal model, the discrete optimized potential energy score of each residue was calculated by using MODELLER [30]. The pentameric GABA receptor was created by superimposing the optimal monomer onto the corresponding monomers of the template, followed by energy optimization using MD simulation to remove the steric clashes at the subunits interfaces.

The MD simulation was carried out using the Gromacs 4.0 package [31]. GROMOS96 43a1 force field [32] was used to describe the protein parameters. A rectangular box with a side length of 95.49 Å × 96.65 Å × 101.01 Å was applied for the system, which was neutralized by adding 5 Na^+^ counterions and then solvated in a truncated octahedral box of SPC waters with [33] a margin distance of 10 Å. All bonds involving hydrogen were constrained using the LINCS algorithm [34]. The long-range electrostatic interaction was calculated by using the particle-mesh-Ewald (PME) method [35] with a cutoff of 10.0 Å. All simulations were performed at constant temperature (300 K) and pressure (1 atm) using the Berendsen coupling algorithm [36] under periodic boundary conditions.

Prior to MD simulation, the whole system was subjected to 10,000 steps of energy minimization to relieve the geometric strain and close intermolecular contacts. The minimized system was gradually heated up to and maintained at 300 K with a coupling coefficient of 1.0/ps in a pressure-constant period of 50 ps. Subsequently, a 500 ps Langevin dynamics calculation was performed with a 2 fs time step at constant temperature (300 K) and pressure (1 atm). Finally, the production phase was run for 5 ns with a 2 fs time step. Coordinate trajectories were recorded every 2 ps during the entire simulation process.

### 3.4. Docking

To predict the appropriate interaction of APDs with housefly GABA receptor, molecular docking was performed to dock all compounds into the active site of the GABA receptor using the Surflex module of SYBYL. During Surflex-docking, two parameters, *i.e*., protomol-bloat and protomol-threshold were used to define the protomol, which is a computational representation of the intended binding site where putative ligands are aligned [37]. The putative poses of docked compounds were scored using the Hammerhead scoring function, which also served as an objective function for local optimization of poses [38]. According to each scoring function, the highest-ranking poses for all compounds were aligned together for CoMFA and CoMSIA modeling.

### 3.5. 3D-QSAR Analysis

In CoMFA, the steric and electrostatic interactions of the probe atoms with each atom in the molecule were computed by using the Tripos force field [26] with radius 1 Å and charge +1 in a regularly spaced (2 Å) grid. The steric and electrostatic energy values were truncated at a default value (30 kcal/mol). Five similarity index descriptors of CoMSIA–steric (S), electrostatic (E), hydrophobic (H), hydrogen bond donor (D) and hydrogen bond acceptor (A), were calculated using the same lattice box used in the CoMFA calculations with the standard settings: charge +1, radius 1 Å, hydrophobicity +1, hydrogen bond donating +1, hydrogen bond accepting +1 [37]. CoMSIA similarity indices (*A_F_*) for a molecule *j* with atoms *i* at the grid point *q* are calculated by Equation (1) as follows:

(1) $${A_{F,K}}^{q}(j)=-\sum \omega_{probe,k}\omega_{ik}e^{-{ar}_{iq}^{2}}$$

where *k* represents the steric, electrostatic, hydrophobic, hydrogen-bond donor and acceptor properties; *i* is the summation index over all atoms of the molecule *j* under investigation; *ω_ik_* is the actual value of the physicochemical property k of atom i; *r_iq_* is the mutual distance between probe atom at grid point *q* and atom *i* of the test molecule; *ω_probe,k_* is the value of the probe atom. The attenuation factor αwas set to the default value of 0.3.

Partial least-squares (PLS) method was used to linearly correlate the CoMFA/CoMSIA descriptors to the binding affinity values. The optimal number of PLS components was determined by leave-one-out (LOO) cross-validation method with a cross-validation coefficient (*q^2^)*. Using the optimum number of components, non-cross-validated correlation coefficient (*r^2^*_ncv_) was calculated subsequently. The predictive correlation coefficient *r^2^*_pred_, based on the test set molecules, was calculated by Equation (2) as follows:

(2) $${r^{2}}_{\text{pred}}=1-(\text{PRESS}/\text{SD})$$

where SD is the sum of squared deviations between the biological activities of the test set molecules and the mean activities of the training set molecules, and PRESS is the sum of squared deviation between the actual and predicted activities of the test set molecules. Finally, CoMFA/CoMSIA coefficient maps were generated by interpolation of the pairwise products between the PLS coefficients and the standard deviations of the corresponding CoMFA and CoMSIA descriptor values.

## 4. Conclusion

In this work, the ligand- and receptor-based 3D-QSAR studies on 60 APDs of insect GABA receptor inhibitors are performed using CoMFA and CoMSIA tools. From the resultant models, the reasonable statistical properties prove that the 3D-QSAR models developed in this work are stable and statistically reliable. The resulting contour maps generated from the best CoMFA and CoMSIA models correlate well with the structural and functional features of APDs in the binding site. On the other hand, the three-dimensional model of housefly GABA receptor is generated by homology modeling with the X-ray crystal structure of the glutamate-gated chloride channel receptor as a template. Molecular docking was employed to elucidate the potential binding mode of APDs inhibitors in the housefly GABA receptor, which reveals that some residues, such as A2′ and T6′ in TM2 region of Rdl subunit, play important roles in the inhibitor binding. In conclusion, the good consistency between the 3D-QSAR and docking results proves again the robustness of the 3D-QSAR models and the reasonability of the docking model, which can be utilized in future rational design of novel potential insecticides.

## Figures and Tables

**Table 1:** Statistical results of CoMFA and CoMSIA models.

Parameters	Ligand-Based		Receptor-Based	

	CoMFA	CoMSIA	CoMFA	CoMSIA
*q^2^*^a^	0.60	0.62	0.34	0.55
*r^2^*_ncv_^b^	0.94	0.97	0.81	0.85
*SEE*^c^	0.48	0.32	0.78	0.70
*F*^d^	90.71	126.18	56.61	45.23
*r^2^*_pred_^e^	0.82	0.95	0.85	0.90
*SEP*^f^	1.17	1.22	1.44	1.21
N_c_^g^	6	10	3	5

Field contribution				
S	0.573	0.139	0.533	0.222
E	0.427	0.338	0.467	-
H	-	0.383	-	0.515
D	-	0.059	-	0.091
A	-	0.081	-	0.173

a Cross-validated correlation coefficient using the LOO methods;

b Non-cross-validated correlation coefficient;

c Standard error of estimate;

d Ratio of *r^2^*_ncv_ explained to unexplained = *r^2^*_ncv_/(1 − *r^2^*_ncv_);

e Predicted correlation coefficient for the test set compounds;

f Standard error of prediction;

g Optimal number of principal components.

**Table 2:** Structures and inhibitory activities of all 3-arylpyrimidin-2, 4-diones (APDs) in the dataset.

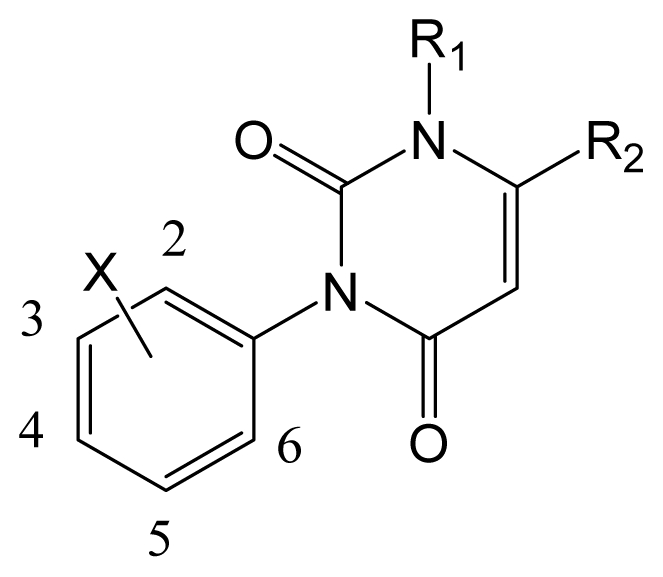									
No	X	R1	R2	p*K*_i_^a^	No	X	R1	R2	p*K*_i_^a^
1	4-Cl	CH3	CF3	5.4	31	3-Cl,4-Cl,5-Cl	CH3	CH2SCH3	4.6
2	3-Cl	CH3	CF3	5.8	32	3-Cl,4-Cl,5-Cl	CH3	COOH	4.0
3	3-Cl,5-Cl	CH3	CF3	6.8	33	3-Cl,4-Cl,5-Cl	CH3	CN	6.2
4#	3-Cl,4-Cl	CH3	CF3	6.6	34	3-Cl,4-Cl,5-Cl	CH3	CH2CH2CF3	6.4
5#	2-Cl,3-Cl	CH3	CF3	6.6	35	3-Cl,4-Cl,5-Cl	CH3	CH2CH2CH3	6.2
6#	2-Cl,6-Cl	CH3	CF3	5.6	36	3-Cl,4-Cl,5-Cl	CH3	CF2CF2CF2CF3	5.0
7#	3-Cl,4-Cl,5-Cl	CH3	CF3	8.0	37	3-Cl,4-Cl,5-Cl	CH3	2-*F*-phenyl	4.0
8	2-Cl,3-Cl,5-Cl	CH3	CF3	7.4	38#	3-Cl,4-Cl,5-Cl	CH3	4-*F*-phenyl	5.0
9	2-Cl,4-Cl,6-Cl	CH_3_	CF_3_	5.2	39#	3-Cl,4-Cl,5-Cl	CH_3_	Cl	5.6
10	2-Cl,3-Cl,6-Cl	CH_3_	CF_3_	7.6	40#	4-Cl	NH_2_	CF_3_	6.7
11	2-Cl,4-Cl,5-Cl	CH_3_	CF_3_	7.1	41#	2-Cl,4-Cl	NH_2_	CF_3_	7.5
12	-H	CH_3_	CF_3_	4.0	42	3-Cl,5-Cl	NH_2_	CF_3_	7.7
13	3-Cl,4-Cl,5-Cl	CH(CH_3_)_2_	CF_3_	6.5	43#	3-Cl,4-Cl	NH_2_	CF_3_	7.7
14#	3-Cl,4-Cl,5-Cl	CH_2_C(CH_3_)_3_	CF_3_	6.2	44	2-Cl,4-Cl,5-Cl	NH_2_	CF_3_	7.5
15	3-Cl,4-Cl,5-Cl	CH_2_CF_3_	CF_3_	7.2	45#	2-Cl,4-Cl,6-Cl	NH_2_	CF_3_	7.5
16	3-Cl,4-Cl,5-Cl	CH_2_COCH_3_	CF_3_	3.8	46	2-Cl,6-Cl	NH_2_	CF_3_	7.1
17	3-Cl,4-Cl,5-Cl	CH_2_CN	CF_3_	7.4	47	3-Cl	NH_2_	CF_3_	6.5
18	3-Cl,4-Cl,5-Cl	CH_2_Ph	CF_3_	3.9	48	2-Cl,3-Cl,4-Cl	NH_2_	CF_3_	8.5
19	3-Cl,4-Cl,5-Cl	CH_2_CH_2_OCH_3_	CF_3_	6.6	49	-H	NH_2_	CF_3_	5
20	3-Cl,4-Cl,5-Cl	CH_2_CH_2_CH_2_CH_3_	CF_3_	4.0	50	3-Cl,4-I,5-Cl	NH_2_	CF_3_	9.3
21#	3-Cl,4-Cl,5-Cl	H	CF_3_	5.5	51	3-Cl,4-SOCH_3_,5-Cl	NH_2_	CF_3_	7.5
22	3-Cl,4-Cl,5-Cl	NH_2_	CF_3_	8.8	52	3-Cl,4-SCH_3_,5-Cl	NH_2_	CF_3_	8.7
23	3-Cl,4-Cl,5-Cl	NHCOCH_3_	CF_3_	5.3	53#	3-Cl,4-Ph,5-Cl	NH_2_	CF_3_	8.9
24	3-Cl,4-Cl,5-Cl	CH_3_	CH(CH_3_)_2_	6.3	54	3-Cl,4-OCH_3_,5-Cl	NH_2_	CF_3_	8.5
25	3-Cl,4-Cl,5-Cl	CH_3_	CF_2_CF_2_ CF_3_	7.3	55	3-Cl,4-(2-thienyl),5-Cl	NH_2_	CF_3_	9.1
26	3-Cl,4-Cl,5-Cl	CH_3_	CH_2_O CH_3_	5.1	56#	3-Cl,4-N(CH_3_)_2_,5-Cl	NH_2_	CF_3_	8.8
27	3-Cl,4-Cl,5-Cl	CH_3_	Ph	4.0	57	3-Cl,4-OCH(CH_3_)_2_,5-Cl	NH_2_	CF_3_	9.0
28	3-Cl,4-Cl,5-Cl	CH_3_	CH_2_SO_2_ CH_3_	42	58	3-Cl,4-Br,5-Cl	NH_2_	CF_3_	9.3
29	3-Cl,4-Cl,5-Cl	CH_3_	CH_3_	4.6	59	3-Cl,4-CF_2_CF_3_,5-Cl	NH_2_	CF_3_	8.8
30	3-Cl,4-Cl,5-Cl	CH_3_	C(CH_3_)3	7.5	60#	3-F,4-CF_3_,5-F	NH_2_	CF_3_	8.7

# Test set compound;

a obtained from ^3^[H] EBOB binding assay using housefly head tissue.
